# Importance of Bmal1 in Alzheimer's disease and associated aging‐related diseases: Mechanisms and interventions

**DOI:** 10.1111/acel.13704

**Published:** 2022-09-03

**Authors:** Rongping Fan, Xuemin Peng, Lei Xie, Kun Dong, Delin Ma, Weijie Xu, Xiaoli Shi, Shujun Zhang, Juan Chen, Xuefeng Yu, Yan Yang

**Affiliations:** ^1^ Department of Endocrinology, Tongji Hospital, Tongji Medical College Huazhong University of Science and Technology Wuhan China; ^2^ Branch of National Clinical Research Center for Metabolic Diseases Wuhan China; ^3^ Department of Neurosurgery, Tongji Hospital, Tongji Medical College Huazhong University of Science and Technology Wuhan China

**Keywords:** Alzheimer disease, ARNTL transcription factors, atherosclerosis, diabetes mellitus, type 2, osteoarthritis, Parkinson disease

## Abstract

With the aging world population, the prevalence of aging‐related disorders is on the rise. Diseases such as Alzheimer's, type 2 diabetes mellitus (T2DM), Parkinson's, atherosclerosis, hypertension, and osteoarthritis are age‐related, and most of these diseases are comorbidities or risk factors for AD; however, our understandings of molecular events that regulate the occurrence of these diseases are still not fully understood. Brain and muscle Arnt‐like protein‐1 (Bmal1) is an irreplaceable clock gene that governs multiple important physiological processes. Continuous research of Bmal1 in AD and associated aging‐related diseases is ongoing, and this review picks relevant studies on a detailed account of its role and mechanisms in these diseases. Oxidative stress and inflammation turned out to be common mechanisms by which Bmal1 deficiency promotes AD and associated aging‐related diseases, and other Bmal1‐dependent mechanisms remain to be identified. Promising therapeutic strategies involved in the regulation of Bmal1 are provided, including melatonin, natural compounds, metformin, d‐Ser2‐oxyntomodulin, and other interventions, such as exercise, time‐restricted feeding, and adiponectin. The establishment of the signaling pathway network for Bmal1 in aging‐related diseases will lead to advances in the comprehension of the molecular and cellular mechanisms, shedding light on novel treatments for aging‐related diseases and promoting aging‐associated brain health.

## Graphical abstract

- Bmal1 deficiency promotes Alzheimer's disease, Parkinson's disease, type 2 diabetes mellitus, atherosclerosis, hypertension, and osteoarthritis.
- These aging‐related diseases are contributors to Alzheimer's disease.
- Oxidative stress and inflammation may be the common trigger when Bmal1 is lost in the brain, pancreas, blood vessels, and articular cartilage.

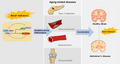

**Abbreviations**
AD: Alzheimer's disease
ADAMTS: a disintegrin and metalloproteinase with thrombospondin motif
AMPK: adenosine 5′‐monophosphate (AMP)‐activated protein kinase
AS: atherosclerosis
BBB: blood–brain barrier
bHLH‐PAS: helix–loop–helix/Per‐ARNT‐SIM
BMAL1: brain and muscle Arnt‐like protein‐1
BMP: bone morphogenetic protein
CA: cornu ammonis
cGAS‐STING: cyclic GMP‐AMP synthase (cGAS) and stimulator of interferon genes
CLOCK: circadian locomotor output cycles kaput
CRY: cryptochrome
EndMT: endothelial‐to‐mesenchymal transition
ER: endoplasmic reticulum
ERK: extracellular signal‐regulated kinase
FoxOs: forkhead box O
GABA: gamma‐aminobutyric acid
GLP‐1R/GCGR: glucagon‐like peptide‐1 receptor/glucagon receptor
GSIS: glucose‐stimulated insulin secretion
GST: glutathione transferase
GTP: guanosine triphosphate
HAECs: human aortic endothelial cells
HIF1α: hypoxia‐inducible factor 1alpha
IAPP: islet amyloid polypeptide
iBRB: inner blood–retina barrier
IL: interleukin
MAPK: mitogen‐activated protein kinases
MMPs: matrix metalloproteinases
MuSC: muscle stem cell
NAD (+): nicotinamide adenine dinucleotide oxidase
NAMPT: nicotinamide phosphoribosyltransferase
NFATC: nuclear factor of activated T cells
NF‐κB: nuclear factor‐kappaB
NMN: nicotinamide mononucleotide
NR: nicotinamide riboside
NRF2: nuclear factor erythroid 2‐related factor 2
OA: osteoarthritis
Oxy: D‐Ser2‐oxyntomodulin
PD: Parkinson's disease
PER: period
PGC‐1α: peroxisome proliferator‐activated receptor‐gamma coactivator‐1alpha
PI3K/AKT/GSK3β: phosphatidylinositol‐3‐kinase/protein Kinase B/glycogen synthase kinase‐3beta
P. gingivalis: porphyromonas gingivalis
REV‐ERB: reverse erythroblastosis virus
RPE: retinal pigment epithelium
RORα: retinoid‐related orphan receptor‐α
ROS: reactive oxygen species
SCN: suprachiasmatic nucleus
SIRT1: Sirtuin1
SMAD: Drosophila similar to mothers against decapentaplegic
STAT: signal transducer and activator of transcription
TGF‐β: transforming growth factor‐beta
TMJ‐OA: temporomandibular joint osteoarthritis
TRF: time‐restricted feeding
TTFL: transcriptional–translational feedback loop
T2DM: type 2 diabetes mellitus
VEGF: vascular endothelial‐derived growth factor
WAT: white adipose tissue

## 1 INTRODUCTION

Aging, a complex biological process, is characterized by decreased physiological function with increased age; often aging is associated with increased susceptibility to diseases, including type 2 diabetes mellitus (T2DM), cardiovascular and musculoskeletal diseases, and neurological diseases such as Alzheimer's disease (AD) and Parkinson's disease (PD) (Zhuang et al., 2019). AD is the foremost cause of impaired cognition in the elderly and has become the fifth leading cause of death worldwide (Cortes‐Canteli & Iadecola, 2020). Studies have found that many aging‐related diseases such as PD (Cibulka et al., 2022), diabetes mellitus, atherosclerosis (AS) (Xie, Shi, et al., 2020), hypertension (Shih et al., 2018), osteoarthritis (OA) (Innes & Sambamoorthi, 2020), and age‐related macular degeneration (AMD) (Wen et al., 2021) are closely related to AD, which may be comorbidities or risk factors for AD. This important commonality is why AD and these diseases are explored in the present study. It is known that PD and AD are common neurologic diseases of old age (Fang et al., 2018). Diabetes mellitus, hypertension, and OA have been reported to be common comorbidities in AD (Kao et al., 2021; Wang, Wu, et al., 2018). AS (Xie, Shi, et al., 2020), and AMD (Wen et al., 2021) had an increased risk of their condition advancing to AD. According to the World Health Organization (WHO) estimates, by 2050, the proportion of the global population with age > 60 years will ascend to 22% (Mahjoob & Stochaj, 2021). Emerging evidence now suggests an increased trend of patients with AD and associated aging‐related diseases. For example, in the United States, the number of patients aged ≥65 years with AD and related dementias is growing and is projected to reach 13.9 million by 2060 (Matthews et al., 2019). The number of adults aged ≥65 and 65–99 years with diabetes mellitus is expected to increase to 0.253 and 1.42 billion, respectively, over the next 23 years globally (Bellary et al., 2021); nearly half of these adults are expected with T2DM. These epidemiological studies indicate a serious threat to global health with reduced quality of life of older adults amidst increased risk of AD and associated aging‐related diseases.

Brain and muscle Arnt‐like protein‐1 (Bmal1), one of the families of basic helix–loop–helix/Per‐ARNT‐SIM (bHLH‐PAS) domain‐containing transcription factors (Majumdar et al., 2017), is the core regulator of the circadian clock, driving rhythmic expression of circadian clock genes. Bmal1 is essential for regulating the circadian rhythms and maintaining the physiological functions of cells and organs. Evidence indicates that the deletion of *Bmal1* can accelerate aging. For example, symptoms of premature aging were observed in Bmal1 deletion animal models, such as organ atrophy, sarcopenia, and cataract; these animals also had shorter lifespans (Kondratov et al., 2006). During natural aging, Bmal1 expression attenuation was noticed in animal models (Duncan et al., 2013). These studies show that the decreased expression of Bmal1 is closely associated with aging. Alterations of Bmal1 during aging, along with other systemic stimulators such as hormones and the changed microenvironment of tissues, may have differential impacts on the brain and peripheral tissues, promoting the progression of aging‐related diseases. The underlying mechanisms of the possible role of Bmal1 dysfunction in AD and associated aging‐related diseases remain unclear; increased understanding of effects of abnormal Bmal1 expression may give rise to the evolvement of new diagnostic and therapeutic approaches for better management of these diseases.

To provide an integrated picture of the role and mechanism of Bmal1 in AD and associated aging‐related diseases, we performed a comprehensive search in PubMed and Google Scholar for relevant studies. Based on existing evidence from the in vivo, in vitro, and clinical studies, we tried to summarize Bmal1‐dependent mechanisms in AD and associated aging‐related diseases and pay attention to therapeutic interventions involved in the regulation of Bmal1.

## 2 THE BIOLOGICAL FUNCTION OF Bmal1

**FIGURE 1:**
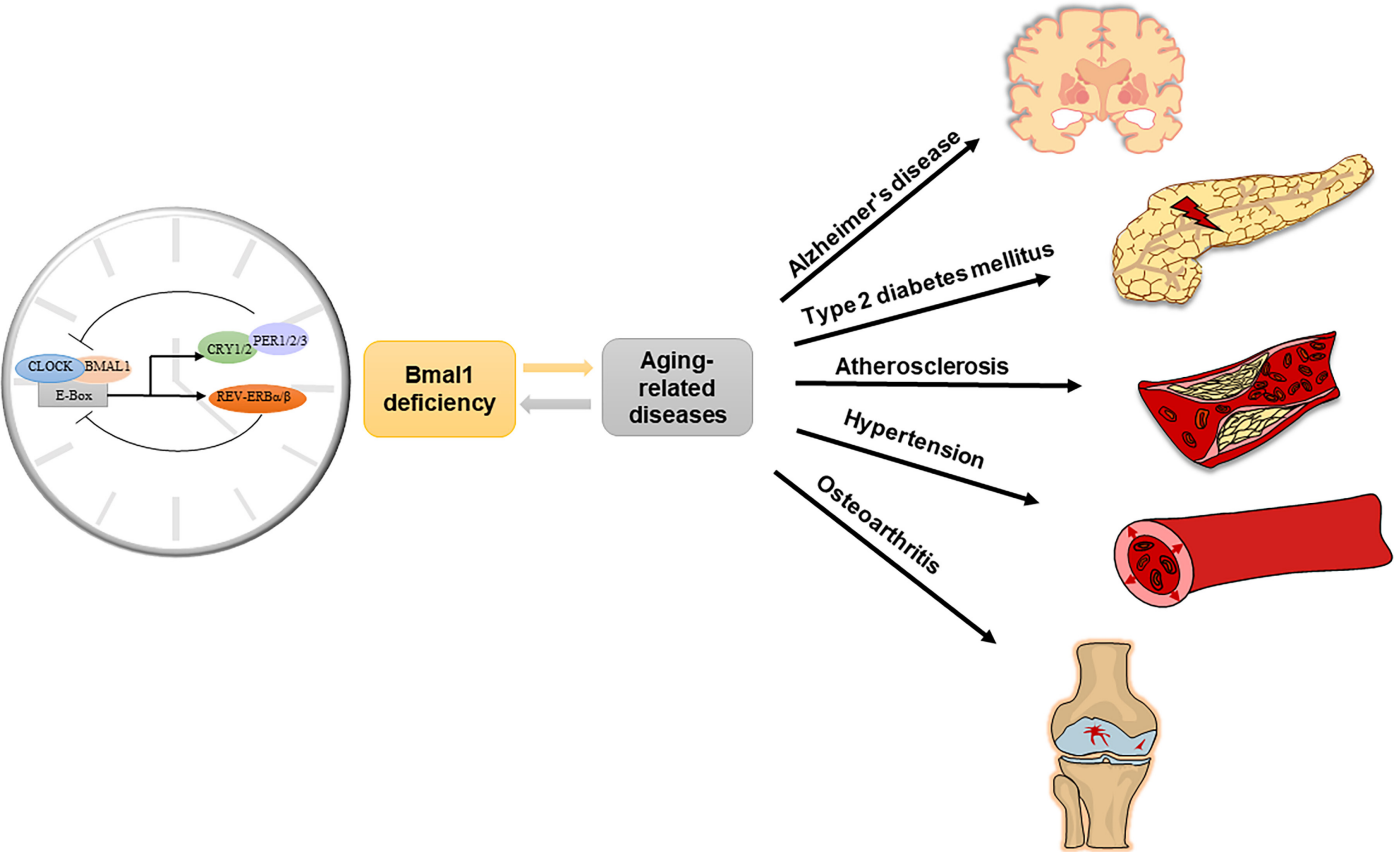
Bmal1 deficiency promotes Alzheimer's disease and associated aging‐related diseases. The mammalian circadian clock exists in nearly all fully differentiated cells, which consists of a transcription‐translation feedback loop (TTFL). The CLOCK‐BMAL1 transcriptional activators bind to the E‐box, driving the expression of CRY‐PER and REV‐ERBs transcriptional repressors. BMAL1 is in an irreplaceable position in TTFL, governing multiple important physiological processes. Bmal1 expression attenuation was observed during aging, and Bmal1 deficiency is an important contributor to aging‐related diseases such as Alzheimer's disease, T2DM, atherosclerosis, hypertension, and osteoarthritis.

*Bmal1*, also called MOP3, is a core driver of the circadian clock in mammals and is considered the only irreplaceable clock gene that regulates rhythmic behaviors (Bunger et al., 2000; Welz et al., 2019). The molecular mechanism, that drives nearly 24 h autonomous circadian oscillations, involves the transcriptional–translational feedback loop (TTFL). Here, Bmal1 and circadian locomotor output cycles kaput (CLOCK) heterodimers form the positive limb that binds to the E‐box motifs and drives the expression of the period (PER1/2/3), cryptochrome (CRY1/2), reverse erythroblastosis virus α (REV‐ERBα), and retinoid‐related orphan receptor‐α (RORα); subsequently, PER and CRY, these two proteins interact and form heterodimers in the cytoplasm, which then translocate to the nucleus to suppress the expression of the positive limb (Richards & Gumz, 2013). Further, REV‐ERBα and RORα, which form additional feedback loops, facilitate and restrain the expression of Bmal1, respectively (Peng et al., 2022). In mammals, the molecular clock based on circadian rhythmicity exists in nearly all fully differentiated cells (Reinke & Asher, 2019). The master clock, situated in the suprachiasmatic nucleus (SCN) of the hypothalamus, receives an immediate projection from the retina via light‐dark cues from the environment (Reinke & Asher, 2019). The SCN synchronizes peripheral clocks located in the non‐SCN brain areas and peripheral tissues such as muscle, liver, adipose tissue, pancreas, and gut through neural, endocrine, temperature, and behavioral signals (Stenvers et al., 2019). The circadian system regulates various physiological processes, including food‐intake behavior, rest‐activity cycle, sleep‐wake cycle, and glucose metabolism (Stenvers et al., 2019). Bmal1 is not only an important regulator of the circadian system but also involves in preserving redox homeostasis (Chhunchha et al., 2020; Xie, Tang, et al., 2020). Furthermore, Bmal1 plays a crucial role in regulating inflammatory responses (Liu et al., 2020), insulin sensitivity (Shi et al., 2013), and mitochondrial functions (E. Li, Li, et al., 2020). *Bmal1* deletion abolishes 24 h activity patterns (Ray et al., 2020), leading to circadian rhythm disorders and aging‐related diseases, such as glycolipid metabolism disorders including T2DM (Marcheva et al., 2010), and neurodegenerative diseases (Musiek & Holtzman, 2016), as presented in Figure 1. Besides, Bmal1 alterations among patients with aging‐related diseases are summarized in Table 1.

**TABLE 1:** Bmal1 alterations among patients with aging‐related diseases

First Author, Year	Participants	age	Cells or tissues	Bmal1 alterations
Hulme et al. (2020)	96 brains of donors who were participants of a large prospective cognitive aging cohort known as The University of Manchester Longitudinal Study of Cognition in Normal Healthy Old Age cohort (UMLCHA)	Age at death = 88.6 ± 5.75 years	Frontal cortex tissues	The positive correlation between DNA methylation at the BMAL1 gene with Braak stage and CERAD score stages, suggests a reduced activity of BMAL1 with increased AD pathology, specifically tau and neurofibrillary tangles
Chen, Peng et al. (2015)	AD patients (*n* = 296) and control subjects (*n* = 423)	AD: 77.84 ± 3.97 years (range: 65 to 85 years) Control: 76.92 ± 3.76 years (range: 65 to 85 years)	Whole blood drawn from the antecubital vein	AD patients showed a higher prevalence of T carriers in BMAL1 rs.2278749 T/C (30.91 vs. 24.82%, *p* < 0.0001) than was observed in controls
Cermakian et al. (2011)	AD patients (*N* = 26, 13 females, 13 males) and controls (*N* = 30, 6 females, 24 males)	AD:79.69 ± 1.64 Control: 74.43 ± 2.16 years	99 samples (29 BNST, 39 cingulate cortex, 31 pineal gland) from 56 donors (26 AD patients, 30 controls)	In AD patients, the phase of the 24‐h harmonic of BMAL1 expression in the pineal and the phases of the 24‐h rhythms of BMAL1 expression in the BNST were significantly different from controls
Yoo et al. (2020)	A total of 12 paraffin embedded brain tissues from 3 donors with AD. A total of 4 paraffin embedded adult normal brain tissues	NA	AD: frontal cortex, occipital cortex, temporal cortex and parietal cortex Control: frontal cortex, occipital cortex, temporal cortex and parietal cortex	The protein levels of BMAL1 were significantly elevated in impaired astrocytes of the cerebral cortex from patients with AD (every individual patient)
Ando et al. (2009)	Patients with type 2 diabetes (*n* = 12) and healthy individual (*n* = 14)	T2DM: 58 ± 6 years Control: 59 ± 6 years	Peripheral leucocytes	T2DM patients expressed significantly lower transcript levels of BMAL1. BMAL1 mRNA levels were inversely correlated with HbA1c levels (r = −0.47, *p* < 0.05)
Pappa et al. (2013)	Gestational diabetes mellitus (GDM) (*n* = 185) and pregnant women with normal glucose tolerance (*n* = 161)	GDM: 33.63 ± 5.14 years Control: 30.71 ± 9.56 years	Peripheral blood leukocytes from 20 GDM and 20 control. Patients are selected according to their polymorphism of Bmal1 gene	Bmal1 is a crucial susceptibility gene for GDM in Greek women. The expression levels of BMAL1 mRNA were significantly lower in GDM patients than in controls
Gunton et al. (2005)	Type 2 diabetic subjects (*n* = 5) and normoglycemic controls (*n* = 7)	Average age was 47 years in both groups. The mean duration of T2DM was 5.8 ± 2.1 years	Diabetic human pancreatic islets	Bmal1 expression was significantly downregulated
Yu et al. (2019)	T2D patients (*n* = 36) and non‐diabetic volunteers (*n* = 14)	T2D: 49.47 ± 7.88 years Control: 45.93 ± 7.16 years	Peripheral blood leucocytes	The BMAL1 mRNA levels were decreased in the diabetic patients. In addition, HbA1c levels were negatively correlated with BMAL1 mRNA levels
Cai et al. (2010)	PD patients (*n* = 17) and age‐matched controls (*n* = 16)	PD: 62.2 ± 2.8 years Control: 57.8 ± 1.1 years	Peripheral leukocytes. Blood samples were collected during 21:00–09:00	Expression of BMAL1 was reduced in PD. The expression levels of BMAL1 in PD patients were correlated with their disease severity and sleep quality
Li et al. (2021)	PD patients (*n* = 326) and healthy controls (*n* = 314)	PD: 67.43 ± 9.71 years Control:66.03 ± 9.24 years	Peripheral blood mononuclear cells (PBMCs)	BMAL1 expression was significantly decreased in PD. The severity of pRBD, the severity of daytime sleepiness, and the self‐reported sleep quality were inversely associated with the expression levels of the BMAL1 gene
Breen et al. (2014)	Parkinson disease cohort (*N* = 239), subgroup of these patients (*n* = 30) and healthy age‐ and sex‐matched controls (*n* = 15)	PD: age at diagnosis = 68 ± 9 years	Peripheral blood	PD patients had a lack of time‐dependent variation in Bmal1 expression
Akagi et al. (2017)	OA patients (*n* = 14) and normal controls (*n* = 14)	OA: 10 females (mean age = 69 years, range:61–82) and 6 males (mean age = 71 years, range: 66–84) Control: 5 females (mean age = 39 years range:26–57) and 18 males (mean age = 30, range:18–44)	Human knee cartilage	BMAL1 mRNA and protein levels were significantly reduced in OA compared with normal cartilage
Dudek et al. (2016)	Non‐OA or mild OA samples (*n* = 6), grade 0/1; grade 2/3, moderate OA samples (*n* = 6); and grade 4, OA (*n* = 6)	Age range, 45–60 years	Human cartilage	The number of BMAL1‐positive chondrocytes and the protein levels of BMAL1 were progressively reduced in OA cartilage with increasing severity compared with the numbers detected in non‐OA human tissue
Wu et al. (2019)	Atherosclerosis patients (*n* = 31) and healthy controls (*n* = 15)	More than 40 years, from both sexes	Plasma from blood specimens	The expression of Bmal1 mRNA was decreased in the plasma of patients with atherosclerosis

Abbreviations: AD, Alzheimer's disease; BMAL1, brain and muscle Arnt‐like protein‐1; BNST, bed nucleus of the stria terminalis; CERAD, Consortium to Establish a Registry for Alzheimer's Disease; GDM, gestational diabetes mellitus; OA, osteoarthritis; PD, Parkinson's disease; pRBD, rapid eye movement sleep behavior disorder; T2D, type 2 diabetes.

## 3 THE ROLE AND MECHANISM OF Bmal1 IN AD

**FIGURE 2:**
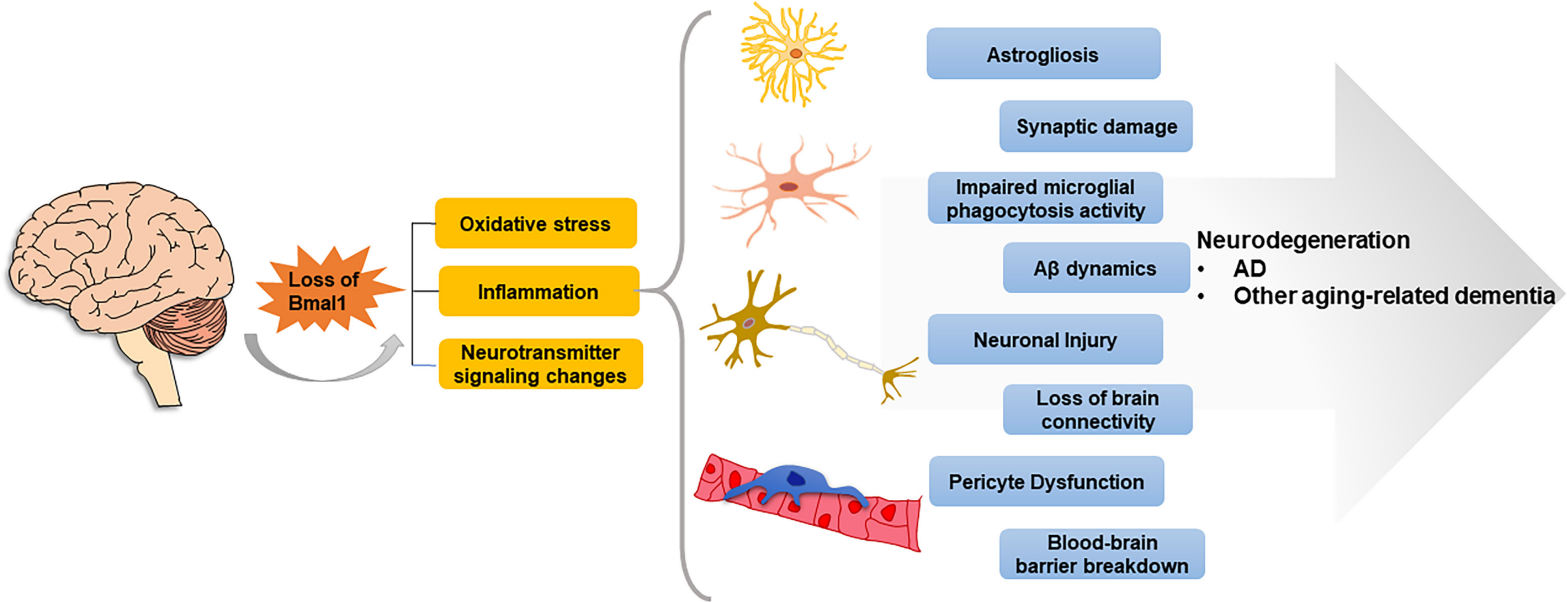
Loss of Bmal1 contributes to the neurodegeneration associated with Alzheimer's disease and other aging‐related dementia. The loss of Bmal1 in the brain has adverse effects on the astrocytes, microglia cells, neurons, and pericyte cells, leading to an increased level of oxidative stress and inflammation, as well as neurotransmitter signaling changes. The above alterations may play a contributory role in astrogliosis, impaired microglial phagocytosis activity, neuronal injury, and pericyte dysfunction, further leading to synaptic damage, Aβ dynamics, loss of brain connectivity, and disruption of the blood–brain barrier, which constitutes the pathological basis of Alzheimer's disease and other aging‐related dementias.

AD is the most prevalent aging‐associated neurodegenerative disorder globally. It is characterized by the accumulation of extracellular β‐amyloid (Aβ) plaques and intracellular hyperphosphorylated tau protein, forming neurofibrillary tangles in the brain. These factors promote the progressive destruction of dendritic spines, synapses, and loss of nerve cells, impaired neurotransmission, progressive isolation of remaining nerve cells, and eventually brain atrophy (Scheltens et al., 2016). The loss of the normal circadian rhythm is a common symptom of AD and is characterized by the increased sleep and awakening during the day and night, respectively (Mattis & Sehgal, 2016). AD‐related circadian dysfunction has been widely explored by disrupting Bmal1 to understand the related mechanism, suggesting an important regulatory role of Bmal1 in AD. The contribution of Bmal1 deficiency to the neurodegeneration associated with AD and other aging‐related dementia is depicted in Figure 2.

### 3.1 Abnormal expression of Bmal1 promoted Aβ accumulation and hyperphosphorylation of tau protein

Many studies have shown that the Bmal1 function loss and Aβ deposition are mutually promoting scenarios. Aβ can incur Bmal1 degradation in AD mouse models (Song et al., 2015). Further, the aberrant expression of Bmal1 mRNA and protein in the mouse hippocampus can be induced by Aβ (Wang, Lv, et al., 2018), while it has also been reported that the Bmal1 loss can in turn accelerates the amyloid plaques accumulation (Kress et al., 2018). These studies indicated that the altered Bmal1 levels might be the cause or a consequence of AD pathology. The effect of Bmal1 deletion on Aβ dynamics and amyloid plaque deposition depends on the scope of the deletion; the global deletion including the SCN generates an aberrant diurnal fluctuation of Aβ in the hippocampal interstitial fluid and speeds up the amyloid plaque accumulation, whereas targeted *Bmal1* deletion in the hippocampus does not alter interstitial fluid Aβ rhythms (Kress et al., 2018). Recently, it has also been found that the loss of Bmal1 from astrocytes does not interpret the increased plaque burden in whole‐brain Bmal1‐knockout mice, suggesting that the status of astrocyte activation in AD brains has complex effects; further studies are needed to explore its non‐Aβ‐associated contribution in AD (McKee et al., 2022). Interestingly, the inhibition of REV‐ERBs can increase the transcription of BMAL1 and enhance microglial Aβ phagocytic activity in AD animal models, which may be a strategy to clear and reduce the amyloid plaque deposition (Lee et al., 2020). In addition, deficiency of Bmal1 may lead to sleep disorders (Akladious et al., 2018; Qiu et al., 2019), preventing the removal of Aβ and increasing the inflammatory cytokines and formation of Aβ plaques (Ettcheto et al., 2019). Furthermore, Bmal1 deletion induced pericyte dysfunction and decreased the integrity of the blood–brain barrier (BBB) in an age‐dependent manner, which promoted the decreased cerebral blood flow rate and accumulation of blood‐derived neurotoxins (Nakazato et al., 2017). Additionally, the link between Bmal1 and tau protein has been confirmed in several studies. Disturbances in normal circadian rhythms also contribute to more disruptive daily oscillations of *Bmal1* and other clock genes and aggravate the hyperphosphorylation levels of soluble tau protein. These observations provide a link between *Bmal1* expression rhythms and AD pathology (Huang et al., 2021). Abnormal tau phosphorylation correlates with alterations in Bmal1 protein levels (Niu et al., 2021), while in patients with AD, methylation of Bmal1, indicating Bmal1 activity reduction, was correlated with tau pathology assessed by CERAD score, night wake, and alterations in cognitive function of some dimensions (Hulme et al., 2020).

### 3.2 Bmal1 deficiency aggravated neuropathology and synaptic degeneration

Astrocytes are the most abundant cell type in the central nervous system (CNS), having close structural and functional interactions with neurons and synapses (Dallérac et al., 2018). Further, reactive astrogliosis is a hallmark of AD (Reichenbach et al., 2019). Since it is difficult to select regulatory elements (to target *Bmal1* for deletion) in all the astrocytes in vivo using the available tools, the deletion of *Bmal1* in astrocytes in many studies refers to either the Bmal1 deficiency in astrocytes specific to certain brain regions such as the SCN, or the astrocytes in the brain of *Bmal1* global knockout mice (Barca‐Mayo et al., 2017). Mounting evidence shows that the influence of Bmal1 deficiency on astrocytes is closely related to pathological factors that promote AD. In an in vitro study, *Bmal1* ^−/−^ astrocytes showed a changed morphology (shorter actin filaments), lower expression of cortactin, and lower levels of Rho‐GTP, impairing the actin cytoskeleton dynamics and formation of the distal astrocyte processes which led to an adverse effect on the synaptic integrity (Ali et al., 2020). In vivo, very fine astrocytic processes were lacking, and the synaptic coverage of astrocytes in CA3 was reduced in *Bmal1* ^−/−^ mice (Ali et al., 2020). These altered synaptic functions are related to cognitive deficits caused by chronodisruption. In addition, the deletion of *Bmal1 in* astrocytes affects neurons; astrocytic Bmal1 is essential to prevent the accumulation of extracellular gamma‐aminobutyric acid (GABA) that mediates astrocyte‐to‐neuron communication (McKee et al., 2022). A study found that the administration of GABA receptor antagonists restored the cognitive functions of *Bmal1*cKO mice, demonstrating that the Bmal1 deficiency in astrocytes may be associated with significant inhibition of learning and memory‐related circuits through altered GABA levels (Barca‐Mayo et al., 2017). Moreover, astrocyte‐specific *Bmal1* deletion promotes neuronal death and astrogliosis and induces inflammatory gene expression in vitro (Lananna et al., 2018). Consistently, inflammatory gene expression and astrocyte activation are also induced in vivo (Lananna et al., 2018). Additionally, when *Bmal1* is abolished in both neurons and astrocytes, these mice show much more remarkable astrogliosis in the whole brain. This particular study also demonstrated that Bmal1 regulates astrogliosis mainly via a cell‐autonomous mechanism mediated by altered glutathione transferase‐mediated protein glutathionylation (Lananna et al., 2018). Musiek et al. found that when *Bmal1* was partially knockdown in primary neurons, these cells exhibited spontaneous neurodegeneration, revealing another cellular mechanism by which diminished Bmal1 expression contributed to neurodegenerative diseases (Musiek et al., 2013); further, Bmal1 deletion contributes to the impaired structure and dysfunction of synapses, as well as damaged cortical functional connectivity in brain regions that are seriously influenced in animal models of brain aging and AD (Musiek et al., 2013).

### 3.3 Signaling pathway for Bmal1 deficiency in AD

**FIGURE 3:**
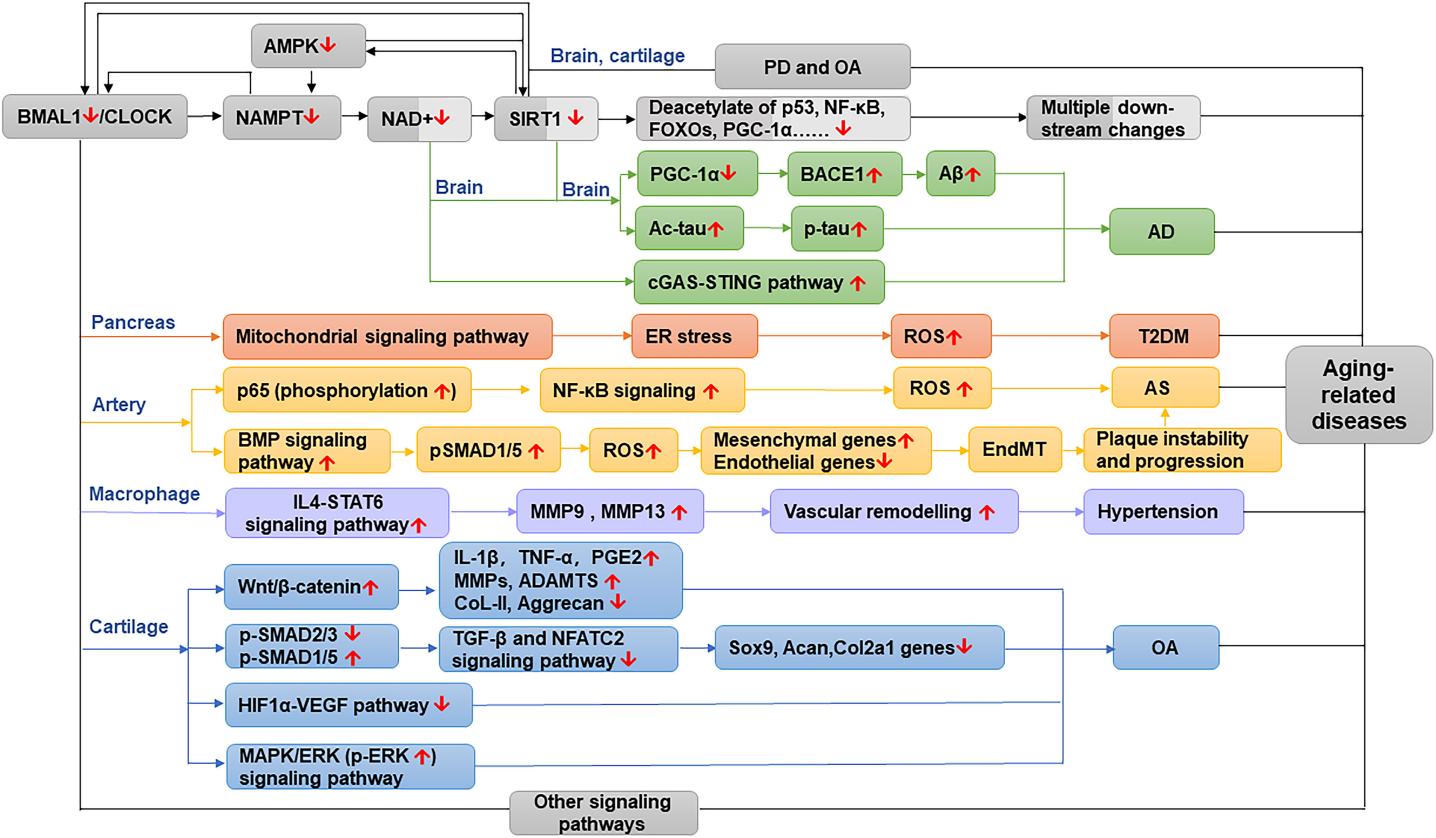
Signaling pathways related to Bmal1 deficiency in AD and associated aging‐related diseases. Dark gray represents the direct link from Bmal1 to NAD+ and SIRT1. Light gray represents the indirect link between Bmal1 to NAD+ and SIRT1. Direct evidence suggested that the SIRT1‐BMAL1 pathway was involved in PD and OA, and indirect evidence indicated that the SIRT1‐BMAL1 pathway may involve in other aging‐related diseases such as AD. Bmal1 deficiency in the pancreas contributed to T2DM via accumulated ROS mediated by the mitochondrial signaling pathway. In the artery, ROS accumulation was aggravated by Bmal1 deficiency through NF‐κB signaling and BMP‐mediated signaling, leading to the progression of AS. Bmal1 loss in macrophages enhanced hypertensive vascular remodeling in hypertension through the IL4‐STAT6 signaling pathway. Bmal1 deficiency in cartilage exacerbated OA through interaction with multiple signaling pathways, which included the Wnt/β‐catenin signaling pathway, TGF‐β and NFATC2 signaling pathway, HIF1α‐VEGF pathway, and MAPK/ERK signaling pathway. There may exist other potential Bmal1‐dependent signaling pathways involved in aging‐related diseases.

Nicotinamide phosphoribosyltransferase (NAMPT) expression is controlled by the BMAL1/CLOCK complex (Ramsey et al., 2009); therefore, the inhibition of nicotinamide adenine dinucleotide (NAD+) biosynthesis is closely associated with Bmal1 deficiency. Sirtuin1 (SIRT1) is an NAD+ ‐dependent enzyme that regulates important physiological process, such as glucose metabolism, mitochondrial homeostasis (Bonkowski & Sinclair, 2016), and immune responses (Chang & Guarente, 2014). It has been reported that the expression of SIRT1 diminishes with the aging (Chen, Zhou, et al., 2020). The correlation of SIRT1 with the molecules participating in various signaling networks (NF‐κB, P53, PGC1α, and FoxOs) has been comprehensively reviewed in the literature (Chen, Zhou, et al., 2020; Gomes et al., 2018). SIRT1 is a positive regulator of *Bmal1* and *clock* genes in SCN (Chang & Guarente, 2013). The results of multiple evidence have indicated that the NAD+/SIRT1 dysfunction contributes to AD (Julien et al., 2009; Koo et al., 2017; Lautrup et al., 2019). Mechanistic studies of the aging brain in PD also showed the involvement of the BMAL1/SIRT1 pathway (Wang, Lv, et al., 2018). Furthermore, NAD+ depletion‐mediated activation of cyclic GMP‐AMP synthase and stimulator of interferon genes (cGAS‐STING) have been reported to play an indispensable role in neuroinflammation and cellular senescence in AD (Hou, Wei, et al., 2021). However, the evidence of pathological progression of AD caused by NAD+ decline caused directly by Bmal1 deficiency is not available, and further studies are needed to reveal the underlying mechanism. Thus, we describe this indirect link in light gray in Figure 3.

## 4 THE ROLE AND MECHANISM OF Bmal1 IN AD‐ASSOCIATED AGING‐RELATED DISEASES

### 4.1 The role and mechanism of Bmal1 in T2DM

T2DM is a prevalent chronic metabolic disease characterized by hyperglycemia, relative insulin deficiency, insulin resistance, and pancreatic beta‐cell dysfunction (Yaribeygi et al., 2020). Bmal1 appears to be highly relevant to the pancreatic islet's function, regulation of insulin secretion, and glucose metabolism. For instance, it was suggested that the expression of Bmal1 in beta cells is a feature of postnatal islet cell maturation and contributes to the establishment of circadian control of glucose‐stimulated insulin secretion (GSIS) (Rakshit et al., 2018). Mice adopt a high‐fat diet undergo compensatory increased beta‐cell mass to preserve normal beta‐cell function and glucose homeostasis (Rakshit et al., 2016); however, this response is diminished in mice with beta‐cell‐specific deletion of Bmal1; increased apoptosis was observed additionally, suggesting a regulating role of Bmal1 in beta‐cell compensatory proliferation and function (Rakshit et al., 2016). Furthermore, Bmal1 in the hypothalamic paraventricular nucleus was significant in regulating insulin secretion and glucose tolerance by regulating the expression and release of vasopressin, a humoral factor that stimulates insulin secretion (Nakata et al., 2021).

Disruption of *Bmal1* in beta cells plays a notable role in the onset of diabetes. Marcheva et al. suggested that pancreas‐specific *Bmal1* KO mice displayed reduced glucose tolerance and lessened pancreatic islets size, proliferation, and insulin release that worsened with age (Marcheva et al., 2010). Lee et al. found that deletion of *Bmal1* in beta cells results in cell dysfunction and diabetes; this was owing to the increased reactive oxygen species (ROS) accumulation, consequent mitochondrial uncoupling, and decreased antioxidant regulatory factor (Lee et al., 2018, 2013). Another study found that the loss of *Bmal1* can impair beta‐cell function through the mitochondrial signaling pathway, as disruptive mitochondrial morphologies such as swelling, fracture, and disappearance of mitochondrial cristae can be observed in *Bmal1*‐deficient beta cells (Ye et al., 2020). Importantly, beta‐cell‐specific *Bmal1* overexpression at the mRNA and protein levels enhances islet's circadian clock amplitude as well as GSIS, meanwhile, preventing obesity‐induced glucose intolerance and reducing the cellular oxidative and endoplasmic reticulum (ER) stress (Rakshit & Matveyenko, 2021). These findings indicate that the downregulation of Bmal1 may be related to T2DM by promoting mitochondrial dysfunction and oxidative stress in beta cells. Genetic variations in Bmal1 have also been linked with susceptibility to T2DM in humans (Pappa et al., 2013; Woon et al., 2007). Consistently, patients with T2DM and animal models of T2DM show disrupted Bmal1 expression in several tissues. For example, downregulated Bmal1 expression was found in diabetic human islets and peripheral blood leukocytes from T2DM patients (Gunton et al., 2005; Yu et al., 2019). Decreased Bmal1 expression in T2DM rats was associated with suppressed bone marrow mesenchymal stem cell osteogenesis (Li et al., 2017). Dampened oscillations of *Bmal1* were found in diabetic mice aortas (Su et al., 2008). Decreased level of Bmal1 protein was found in white adipose tissue (WAT) of db/db mice (Caton et al., 2011). Overall, the findings have revealed that Bmal1 expression is impaired in T2DM.

### 4.2 PD

PD is the second most common age‐related neurodegenerative disorder and is manifested through the motor and non‐motor symptoms (Wang, Lv, et al., 2018). Many clinical manifestations of PD suggest circadian rhythm dysfunction, such as disturbances of rest and activity cycles, aberrant blood pressure fluctuation patterns, and an abnormal rhythm of melatonin secretion (Wang, Lv, et al., 2018). These observations indicate that disrupted circadian rhythm is one of the most common non‐motor symptoms in PD. The clinicopathological features of PD are characterized by the dopaminergic neuronal loss in the substantia nigra pars compacta and the formation of Lewy bodies and neuritis (Gómez‐Benito et al., 2020). Evidence in human studies and animal models showed that decreased level of Bmal1 is involved in PD. Relatively lower Bmal1 levels were found in leukocytes of PD patients, which may be a reflection of the severity of PD (Cai et al., 2010). Bmal1 expression levels in the peripheral blood mononuclear cells and plasma melatonin levels were decreased in patients with PD (Li et al., 2021). Moreover, a lack of time‐dependent variation in Bmal1 expression was observed (Breen et al., 2014). One potential explanation for the alteration of Bmal1 expression is that dopamine can regulate the activity of the BMAL1/CLOCK complex, and hence, a lack of dopamine in PD affects this central component of the molecular clock (Breen et al., 2014). After melatonin treatment, the levels of Bmal1 were increased in PD patients (Delgado‐Lara et al., 2020), reinforcing a close link between Bmal1 and PD. In a rat model of PD, the expression of Bmal1 in the striatum was significantly lower than that in the sham group at some timepoints (Yang et al., 2021). Further, in another rat model of PD induced by lipopolysaccharide combined with rotenone, the mRNA and protein expressions of Bmal1 also decreased (Li et al., 2019).

Additionally, Bmal1 was shown to be involved in regulating PD pathogenesis. In the 1‐methyl‐4‐phenyl‐1,2,4,5‐tetrahydropyridine‐induced PD mouse model, inactivation of Bmal1 can lead to distinct motor dysfunction, dopaminergic neuronal injury in the substantia nigra pars compacta, deficiency in dopamine transmitters, and aggravation of the neuroinflammatory response, suggesting that Bmal1 may exert a beneficial effect on survival of dopaminergic neurons by regulating the microglia‐mediated neuroinflammation response (Liu et al., 2020). The increase in oxidative stress (and loss of antioxidative defense) is critically related to the onset of PD (Johnson et al., 2012); treatment with 6‐hydroxydopamine, in an animal model of PD, caused a reduction and elevation in the levels of SIRTI and acetylated Bmal1, respectively, causing abnormal antioxidative activity. These findings suggest that the SIRT1‐BMAL1 pathway is involved in the regulation of abnormal antioxidative responses in PD (Wang, Lv, et al., 2018).

### 4.3 Atherosclerosis

Several studies indicate a direct link between Bmal1 and atherosclerosis (AS). For instance, Bmal1 downregulation is involved in *Porphyromonas gingivalis* (*P. gingivalis*) induced atherosclerosis by exacerbating the development of oxidative stress (Xie, Tang, et al., 2020). In the indirect co‐culture model of *P. gingivalis* and human aortic endothelial cells, Bmal1 upregulation inhibited monocyte recruitment, reduced the levels of pro‐inflammatory cytokines, and decreased cell apoptosis, demonstrating the antagonistic role of Bmal1 in oxidative stress (Xie, Tang, et al., 2020). The loss of Bmal1 in macrophages could give rise to increased trafficking of Ly6chi monocytes to atherosclerotic lesions, which resulted in an increase in macrophage content and lesion size in the carotid arteries and promoted atherosclerosis (Huo et al., 2017). Bmal1 deficiency in the human carotid aggravated intracellular ROS accumulation and endothelial‐to‐mesenchymal transition through the bone morphogenetic protein‐mediated signaling, demonstrating the central role of Bmal1 loss in atherosclerosis progression (Zhu et al., 2018). Liang et al. found that the upregulated expression of microRNA‐155 (miR‐155) was positively associated with the downregulation of Bmal1 in the aorta, leading to an increased atherosclerotic plaque area and weakened aortic diastolic function, and these changes were reversed after downregulation of miR‐155 or an increase in Bmal1 (Liang et al., 2020). Pan et al. found that both the global and hepatic Bmal1 deficiency enhances atherosclerosis in Apoe−/− mice and demonstrated that Bmal1 is an anti‐atherogenic transcription factor for its important contribution to lipoprotein and cholesterol metabolism (Pan et al., 2016). These studies supported that Bmal1 deficiency plays an important role in AS.

### 4.4 Hypertension

The clock gene Bmal1 is reported to be highly correlated with the etiology of hypertension. Analysis of single‐nucleotide polymorphisms (SNPs) has elucidated that BMAL1 is associated with susceptibility to hypertension (Woon et al., 2007). In a genetic animal model of hypertension, the spontaneously hypertensive (SHR) rats, the expression of Bmal1 was decreased (Tharmalingam et al., 2020). A significant rhythm imbalance of the Bmal1 mRNA levels was found in SHR liver tissues (Hou, Zhang, et al., 2021). Hypertension in db/db mice is associated with attenuated Bmal1 oscillations in the vasculature (Su et al., 2008). Recently, Huo et al. suggested that BMAL1 deletion has a tonic effect on vascular remodeling, leading to the promoted blood pressure increase (Huo et al., 2021). They indicated that loss of Bmal1 enhanced the phosphorylation and nuclear translocation of STAT6 triggered by IL4, which promoted the activation of STAT6 and its target gene transcription, leading to increased expression of MMP9 and MMP13 in the vascular wall, contributing to vascular remodeling (Huo et al., 2021).

### 4.5 Osteoarthritis

Osteoarthritis (OA) is the most widespread degenerative joint disease and is characterized by cartilage degeneration in articulating joints, with a higher risk in older people. The OA disease affects >30 million people in the United States (Alliston et al., 2018). Bmal1 is essential for the development of hard tissues such as bones, cartilage, and teeth (Chen, Tang, et al., 2020). A growing body of studies suggest that *Bmal1* loss can inhibit osteogenesis and promote osteoclastogenesis, playing a significant role in the pathogenesis of OA. Akagi et al. found that Bmal1 is reduced in OA cartilage, and alterations in Bmal1 expression affect transforming growth factor‐beta (TGF‐β) downstream gene expression in chondrocytes, which may accelerate cartilage injury through inflammation‐related pathways (Akagi et al., 2017). Consistently, Dudek et al. found that the expression of Bmal1 was reduced in the articular cartilage of human knees with OA (Dudek et al., 2016). In the *Bmal1*‐cKO mouse model used in their study, targeted *Bmal1* ablation abolished circadian rhythm as well as resulted in progressive degeneration of articular cartilage, which may be related to the disturbance of cartilage homeostasis caused by Bmal1 dysregulation; reduced TGF‐β and NFATC2 pathway signaling was identified in *Bmal1*‐cKO chondrocyte (Dudek et al., 2016). It is known that the increase in matrix metalloproteinases (MMPs) and a disintegrin and metalloproteinase with thrombospondin motif (ADAMTS) downstream induced by the extracellular signal‐regulated kinase (ERK) lead to cartilage decomposition (Ma et al., 2014). Chen et al. observed that Bmal1 expression was decreased in rats with circadian rhythm disruption, along with increased P‐ERK/MMPs/ADAMTS expression (Chen, Zhao, et al., 2020). IL‐6‐induced MMP3/13 and ADAMTS5 upregulation was retracted by Bmal1 overexpression in vitro; temporomandibular joint osteoarthritis (TMJ‐OA) was reversed in Bmal1‐overexpressing rats, suggesting that Bmal1 is involved in the regulation of OA through the mitogen‐activated protein kinase (MAPK)/ERK signaling pathway (Chen, Zhao, et al., 2020). Evidence in vivo and in vitro found that the deletion of *Bmal1* could lead to inhibited chondrocyte proliferation and elevated apoptosis, along with decreased expression of hypoxia‐inducible factor 1alpha (HIF1α) and vascular endothelial‐derived growth factor (VEGF); consistently, the recovery of Bmal1 expression improved the function of chondrocytes (Ma et al., 2019). Moreover, Bmal1 takes part in the regulation of cartilage homeostasis through SIRT1, which is affected by the level of NAD+ oxidase (Yang et al., 2016). Bmal1 also participates in the regulation of matrix synthesis and catabolic metabolism in cartilage and chondrocytes through the Wnt/β‐catenin signaling pathway (Song, Ma, et al., 2021). These findings suggest that Bmal1 deletion of chondrocytes results in disruptive critical pathways that impair cartilage homeostasis and exacerbate a catabolic state, thus resulting in cartilage degradation.

### 4.6 Age‐related macular degeneration

Age‐related macular degeneration (AMD) is a leading cause of visual impairment and severe irreversible blindness and is classified as early‐stage (medium‐sized drusen and retinal pigmentary changes) to late‐stage (neovascular and atrophic) in clinical settings (Mitchell et al., 2018). In patients older than 75 years, the risk of developing early AMD and late AMD is 25% and 8%, respectively, and the number of cases is expected to grow as the population ages (Stahl, 2020). By 2040, the global number of people suffering from AMD is expected to be nearly 300 million (Mitchell et al., 2018). The inner blood–retina barrier (iBRB) is exceedingly dynamic, and claudin‐5, a tight junction protein, is abundantly expressed. Persistent suppression of claudin‐5 induces remarkable retinal pigment epithelium (RPE) cell atrophy (Hudson et al., 2019). Hudson et al. found that disruption of Bmal1 expression results in dysregulated claudin‐5 cycling and adverse effects on the integrity of endothelial cells, suggesting that the Bmal1 dysfunction may strongly implicate in drusen accumulation and subsequent RPE atrophy (Hudson et al., 2019). Therefore, it is necessary to explore a more comprehensive and in‐depth mechanism of Bmal1 in AMD.

## 5 SUMMARY AND DISCUSSION OF THE ROLE AND MECHANISM OF Bmal1 IN AD AND ASSOCIATED AGING‐RELATED DISEASES

The above studies explored the role of Bmal1 in AD and associated aging‐related diseases, which suggested the dysfunction of Bmal1 in many important organs: the brain, pancreas, blood vessels, and articular cartilage. Some ideas can be extracted from these studies. First, oxidative stress may be the common trigger when Bmal1 is lost in these organs, driving the aging‐related loss of function in organs; and inflammation may be another important trigger. This idea was consistent with the main idea of another study discussing oxidative stress in aging and related chronic diseases (Mas‐Bargues et al., 2022). Here are some issues worth considering. At the organ‐tissue level, it would be interesting to know whether Bmal1 loss in one organ affects other organs and whether the start of oxidative stress in one organ may be the trigger of oxidative stress for another organ, contributing to the organ‐organ communication involved in the development of the aging‐related diseases and cognition dysfunction. On a cellular level, it remains unclear whether Bmal1 deficiency in different organs shares a common signal pathway before the initiation of the oxidative stress‐related signal pathway.

Second, evidence showed that Bmal1 was associated with other ischemia‐related diseases such as myocardial infarction (Liu, Xiao, et al., 2021), stroke (Beker et al., 2018), and peripheral vascular disease (Xu et al., 2021), which are highly prevalent in aging populations (Hadjipanayi & Schilling, 2013); and some of these diseases have a close relationship with hypertension and atherosclerosis and may promote AD (Vijayan & Reddy, 2016; Zhang & Luo, 2020). There is an interesting link between vascular damage and Bmal1 deficiency when putting these diseases together, suggesting that a mechanism related to vascular pathology may be a common trigger. Cumulative evidence has demonstrated that the lack of Bmal1 would impair angiogenesis, and endothelial cell functions (Xu et al., 2021), accelerating microvascular and macrovascular damage (Lee et al., 2021). Endothelial dysfunction from the Bmal1‐knockout mice was associated with enhanced superoxide levels and endothelial NO synthase uncoupling in blood vessels (Anea et al., 2012). In part, the mechanisms underlying these impairments may involve oxidative stress and that the Bmal1 exerts on key pathways in endothelial cell signaling.

Third, most studies suggested that decreased level of Bmal1 was a contributor to aging‐related disease; there are also a few studies linking elevated Bmal1 to certain diseases. For instance, the elevated level of Bmal1 was also found in the pineal of streptozotocin‐induced type 1 diabetes (Peschke et al., 2008), astrocytes of the cerebral cortex in AD (Yoo et al., 2020), tissue samples of follicular and papillary thyroid carcinoma (Mannic et al., 2013), indicating that Bmal1 may exhibit a tissue‐specific expression representing an additional regulation. This specific role in AD and associated aging‐related disease and the potential mechanisms remain to be further clarified. Besides, in addition to contributing to the development of aging‐related diseases, the deletion of Bmal1 in specific tissue may offer new treatment strategies for some diseases. For example, microglia‐specific knockdown of Bmal1 improved memory in the animal model, and the authors proposed that microglial Bmal1 may be a potential therapeutic target for metabolic and cognitive disorders related to psychiatric disease (Wang et al., 2021).

**FIGURE 4:**
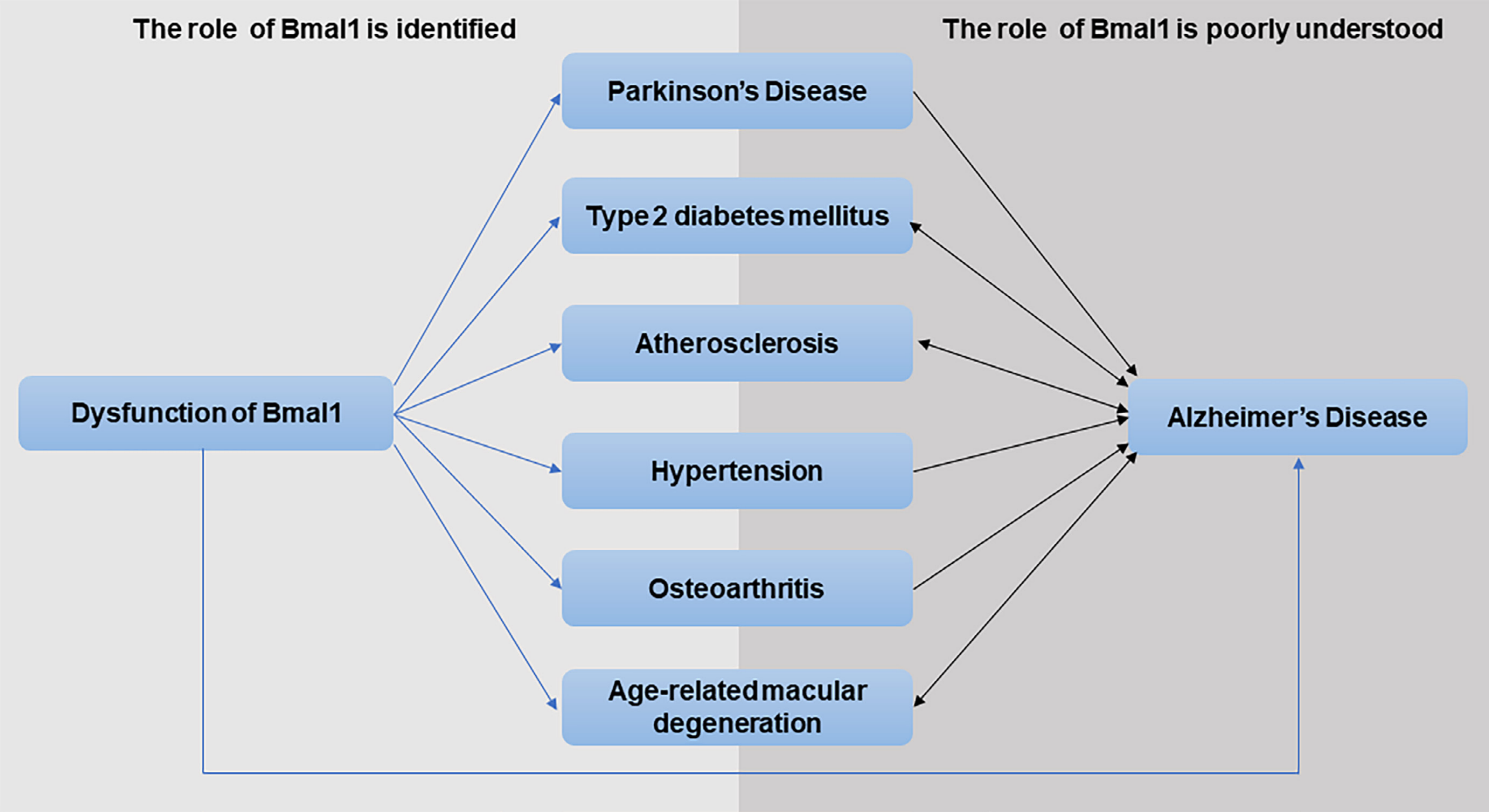
The role of Bmal1 in the mechanisms connecting aging‐related diseases to AD is poorly understood. The role of Bmal1 in AD, PD, T2DM, hypertension, AS, OA and AMD is identified (blue lines). These AD‐associated diseases can also promote AD or have close interaction with AD through numerous pathways (black lines), and the role of Bmal1 in the mechanisms connecting these aging‐related diseases to AD is poorly understood.

The present study identified important role and mechanism of Bmal1 in AD and associated aging‐related diseases such as PD, T2DM, hypertension, AS, OA, and AMD, suggesting that Bmal1 deficiency was implicated in the etiology of these diseases. These AD‐associated diseases can also promote the initiation or progression of AD through numerous pathways, and the pathology of one of the diseases may be a risk factor for another. Thus, it is interesting to explore the role of Bmal1 in this relationship. Unfortunately, there was rare evidence supporting the role of Bmal1 in the contributing effects of AD‐associated diseases on AD. For example, the deposition of islet amyloid polypeptide (IAPP) in the pancreas is one of the pathological hallmarks of T2DM. IAPP was detected in brain tissues and was associated with cognitive decline and AD development; its interaction with Aβ contributed to a synergistic toxic activity (Zhang & Song, 2017). Besides, IAPP was a crucial mediator of tau pathology in AD (Zhang et al., 2022). However, we found no evidence about the role of Bmal1 in the above pathological links, which suggested that the role of Bmal1 in the mechanisms connecting these aging‐related diseases to AD is poorly understood (Figure 4).

## 6 THERAPEUTIC PERSPECTIVES

Therapeutics tailored to regulate molecular Bmal1 could be the key to preventing the progression of aging‐related diseases. Several relevant treatments targeting Bmal1 in aging‐related diseases are discussed in this section and summarized in Table 2. Oxidative stress is a consensus mechanism of aging, and the antioxidant N‐acetyl‐L‐cysteine has been shown to ameliorate signs of premature aging related to Bmal1 deficiency (Kondratov et al., 2009). Antioxidants that boost nuclear factor erythroid 2‐related factor 2 (NRF2) activity are available to rescue the excessive pro‐oxidant and pro‐inflammatory phenotype of *Bmal1* ^*−/−*^ macrophages (Early et al., 2018). Therefore, treatments involving antioxidative effects may have anti‐aging effects, and we focused on those involved in the regulation of Bmal1.

**TABLE 2:** Therapeutic strategies targeting Bmal1 in aging‐related diseases in different models

First Author, Year	Design and subjects	Age^a^	Treatment	Bmal1 alterations	Results
Ikeno and Nelson (2015)	Siberian hamsters	16‐18 was	**Melatonin**, 20 μg, subcutaneous injections 4 h before killed	Increased	Remodeling of hippocampal neurons, altered the synaptic structure in the CA1 region and the dentate gyrus region
Beker et al. (2019)	N2A cells mimic focal cerebral ischemia in vitro	NA	**Melatonin (MEL)**, 1 μM	Increased	Increased cellular survival under normoxic conditions and after oxygen–glucose deprivation
Delgado‐Lara et al. (2020)	A double‐blind, cross‐over, placebo‐controlled RCT, 26 patients with stage 1–3 PD	Median age = 55.5 years	First group: **MEL** (25 mg, twice a day for 3 months)‐ 4 days of washout period ‐Placebo (25 mg, twice a day for 3 months) Second group: Placebo‐ washout period ‐ **MEL**. At the same way	The levels of BMAL1 genes increased from 0.56 to 2.2	The global perception of sleep comfort was increased
Kondratov et al. (2009)	Bmal1−/− mice	4‐45 w	Antioxidant N‐acetyl‐L‐cysteine (NAC), starting from prenatal development, ending in spontaneous death	NA	The age‐dependent weight loss (40 w) was delayed; the incidence of cataracts (phenotype of premature aging) was decreased, and the lifespan was extended
Kim et al. (2021)	APP/PS1 mice, 3 months	19–22 months	Regular diets containing equivalent macronutrients with Purina 5053 with or without 0.1% **nobiletin** (NOB), for 16‐18 months	The mRNA levels of Bmal1 at ZT6 was higher compared with ZT18	The amounts of full‐length APP proteins (APP‐FL) were significantly reduced by NOB at ZT6, but not at ZT18. The expression of clock‐controlled metabolic genes involved in insulin signaling and mitochondrial function were enhanced. The Aβ plaques in the cortex were reduced
He et al. (2016)	C57BL/6J mice, 6 week. HFD for 10 weeks. *Clock* ^*Δ19/Δ19*^ C57BL/6J mice. db/db mice. *db/dbClock* ^*Δ19/Δ19*^ mutant mice	16 w	**Nobiletin** (200 mg/kg body weight) via oral gavage every other day, in the time window of ZT8‐10, for 10 weeks	Increased Bmal1 promoter‐driven luciferase reporter expression with wild‐type, but not mutant	NOB improved glucose and lipid homeostasis in diet‐induced obesity (DIO) mice and *db/db* mice but not in *ClockΔ19/Δ19* C57BL/6J mice, and improved glucose homeostasis in *db/db* ClockΔ19/Δ19 mutant mice
Qi et al. (2017)	Human neuroblastoma SH‐SY5Y cell line	5–8 generations	Pretreated with **tea polyphenols** (TP, purity of >98%) (40 μg/ml) for 12 h and then with H2O2 (100 μM) for 12 h after a washing with PBS	TP functions in a Bmal1‐dependent manner	Result1:TP suppressed H2O2‐triggered apoptosis and loss of cell viability and ameliorated oxidative stress and mitochondrial dysfunction. Result2: The neuroprotective effects of TP were abolished by silencing Bmal1 expression with siRNA
Quezada‐Fernández et al. (2019)	A double‐blind, placebo‐controlled RCT, 20 patients with T2DM, mean age = 53.2 years	Green tea extract (*n* = 10, mean age = 50.2), placebo (*n* = 141, mean age = 56.1)	400 mg of decaffeinated green **tea extract** (polyphenols ≥90%) or placebo for 12 weeks	NA	cAIx75 (75 bpm heart rate adjusted central augmentation index) was decreased. Arterial stiffness was improved
Zhao et al. (2016)	T2DM rats (high‐sucrose‐fat diets for 4 weeks, then intraperitoneally injected with 0.5% STZ solution at the dose of 50 mg/kg weights)	>8 w	LBP‐4a treatment group at high dose (10 mg/kg·d), and the LBP‐4a treatment group at low dose (5 mg/kg·d), intragastric administration for 4 weeks	Increased BMAL1 expression in pancreatic islet cells in high dose group	Blood glucose and insulin were decreased in both high dose and low dose groups. Melatonin was increased in the high dose group. Disruption of pancreatic architecture was meliorated in both high dose (more obvious) and low dose groups
Caton et al. (2011)	Db/db, Db/+ mice, 7 w	8 w	**Metformin**, (250 mg/kg/day for 7 days), by oral gavage	Increased Bmal1 mRNA expression by 2.1‐fold	The BMAL1 protein expression was increased in tWAT of db/db mice. The fasting glucose and fasting insulin were decreased
Alex et al. (2020)	Db/db, Db/+ mice, 8–10 w	10‐12 w	**Metformin** (164 ± 40 mg/kg/day for 14 days)	Increased BMAL1 protein	Body mass and Glycated hemoglobin were decreased. The retinal Kir4.1 level in Müller cells was increased
Wang, Zhao, et al. (2020)	C57BL/6 mice received Aβ31‐35 via hippocampal injection, 9‐11 w	9‐11 w	**D‐Ser2‐Oxyntomodulin** (Oxy) (30 nmol/kg), via hippocampal injection, 15 min before Aβ31‐35 was injected	The rhythmic mRNA and protein expression of Bmal1 in the hippocampus were improved	The Aβ31‐35‐induced disruption of the circadian rhythm (The sleep–wake cycle, free‐running period, locomotor activity, and ratio of the subjective night activity/total activity) was recovered. The abnormal expression of Bmal1 and Per2 was improved in the mice hippocampus and HT22 cells
Erickson et al. (2020)	26 adults with prediabetes and obesity provided with isoenergetic diets	Mean age = 66 years	12‐week **exercise intervention** (5 days per week at 85% of heart rate max on a treadmill for 60 min)	BMAL1 gene expression in skeletal muscle was increased	Body weight, BMI, and total abdominal adiposity were decreased. Exercise capacity and peripheral insulin sensitivity were improved. The fold change in BMAL1 was positively correlated with insulin sensitivity and negatively correlated with BMI
Chaix et al. (2019)	Liver‐specific Bmal1 knockout mice, 12 w	24 w	12 weeks on the **time‐restricted feeding (TRF)**	NA	TRF prevented whole‐body fat accumulation and serum hyperlipidemia
Regmi et al. (2021)	C57BL/6J mice, 8 w, high‐fat diet ad libitum for 4 w	20 w	A further 8 weeks on **TRF**e (initiated at lights off) or TRFd (initiated at 4‐h after lights off)	The amplitude of Bmal1mRNA levels in liver was increased	TRF reduced weight and fat mass (greater reduction in TRFe), improved glucose tolerance, and protected mice from high‐fat diet‐induced hepatosteatosis (no difference in TRFe vs TRFd)

Abbreviations: Aβ, amyloid β‐protein; APP, amyloid precursor protein; BMAL1, brain and muscle Arnt‐like protein‐1; CA, cornu ammonis; DIO, diet‐induced obesity; MEL, melatonin; NAC, antioxidant N‐acetyl‐L‐cysteine; NOB, nobiletin; N2a, neuroblastoma cell lines; Oxy, D‐Ser2‐oxyntomodulin; PD, Parkinson's disease; RCT, randomized controlled trial; STZ, streptozotocin; TP, tea polyphenols; TRF, time‐restricted feeding; T2DM, type 2 diabetes mellitus; WAT, white adipose tissue; ZT, Zeitgeber time.

^a^ The age of mice was sacrificed, or the time point of relative parameters w collected.

### 6.1 Melatonin

Melatonin is an amine hormone and an effective endogenous antioxidant (Tan et al., 2015), synthesized and secreted primarily by the pineal gland under normal light/dark conditions at night. Numerous studies have suggested that melatonin plays an important role in regulating Bmal1 and exhibits beneficial physiological influences in the intervention of aging‐associated diseases such as AD (Li, Zhang, et al., 2020), PD (Delgado‐Lara et al., 2020), T2DM (Abdulwahab et al., 2021), AS (Xie, Tang, et al., 2020), and OA (Hosseinzadeh et al., 2016). Melatonin strengthens the expression of Bmal1 protein and regulates the expression of Bmal1 via PI3K/AKT signaling (Beker et al., 2019). A study showed that melatonin treatment increased Bmal1 expression in the hippocampus, induced rapid remodeling of hippocampal neurons, and altered the synaptic structure in the CA1 and dentate gyrus regions, suggesting that melatonin can regulate the structural changes in hippocampal neurons through the circadian clock molecule Bmal1 (Ikeno & Nelson, 2015). Intermittent administration of melatonin can reset the daily pattern of *Bmal1* expression (Furtado et al., 2020). Furthermore, endogenous and exogenous melatonin ameliorates diabetes and related metabolic dysfunction by regulating insulin secretion and protecting against ROS (Espino et al., 2019). A study reported that melatonin increased the expression of SIRT1‐BMAL1 pathway‐related proteins in a dose‐dependent manner and protected against cerebral ischemia–reperfusion‐induced brain damage in diabetic mice (Liu, Cao, et al., 2021). The above evidence reveals that melatonin may be a prospective therapeutic agent for aging‐related diseases.

### 6.2 Natural compounds

Nobiletin, a citrus flavonoid, was shown to improve cognitive deficits and pathological features and motor and cognitive deficits in AD and PD, respectively, in animal model studies (Nakajima & Ohizumi, 2019). Nobiletin increases diurnal changes in Bmal1 expression and decreases Aβ plaques in the cortex of APP/PS1 mice (Kim et al., 2021). A previous study showed that nobiletin directly binds and activates RORs, while the RORs enhance the activity of nobiletin on Bmal1 transcription. It was also postulated that nobiletin is a natural compound correlated with the enhancement of molecular clock and counteracts metabolic syndrome in a clock‐dependent manner (He et al., 2016). Nobiletin can promote GSIS and prevent ER stress‐induced beta‐cell apoptosis, thereby exhibiting its anti‐diabetic effect (Kaneko et al., 2020). The anti‐diabetic and anti‐inflammatory effects of nobiletin were also determined in an in vitro human model (Nguyen‐Ngo et al., 2020).

Tea polyphenols contain powerful antioxidant properties and ameliorate mitochondrial dysfunction in a Bmal1‐dependent manner, playing an important role in the recovery of neurodegenerative diseases (Qi et al., 2017). Evidence has shown that tea polyphenols also improve glucose metabolism (Egbuna et al., 2021) and memory decline in aging models of rats (Song, Zhang, et al., 2021). Another natural product, LBP‐4a prepared from *Lycium barbarum*, can improve hyperglycemia and insulin resistance by regulating biological rhythms, which are related to the increased expression of Bmal1 in pancreatic islet cells to improve impaired pancreatic islets (Zhao et al., 2016).

### 6.3 Metformin and D‐Ser2‐oxyntomodulin

In db/db mice, decreased AMP‐activated protein kinase (AMPK) activity, NAMPT expression, and SIRT1 expression in WAT were associated with suppressed expression of CLOCK and Bmal1 (Caton et al., 2011). Metformin treatment restored AMPK levels together with evidently increased expression of NAMPT, SIRT1, CLOCK, and Bmal1, suggesting that metformin may regulate glycolipid metabolism partly through AMPK‐NAMPT‐SIRT1‐mediated alterations in the clock components (Caton et al., 2011). Metformin protects against the principal inwardly rectifying potassium‐conducting channel (Kir4.1) in Müller cells; since, Bmal1 plays an intermediary role in the AMPK‐mediated increase in Kir4.1 protein expression, which makes Bmal1 supposed to be a potential therapeutic target to prevent Müller cell dysfunction in diabetic retinopathy (Alex et al., 2020). Although several findings support the therapeutic potential of metformin for age‐related diseases, including AD (Farr et al., 2019), PD (Mor et al., 2020), AS (Wu et al., 2021), and OA (Feng et al., 2020), involvement of Bmal1 is still poorly understood.

D‐Ser2‐oxyntomodulin (Oxy), a new glucagon‐like peptide‐1 receptor/glucagon receptor (GLP‐1R/GCGR) dual receptor agonist, can rescue Aβ31‐35‐induced circadian rhythm disturbance and increase the expression of Bmal1 (Wang, Zhao, et al., 2020). It has also been illustrated that Oxy acts a part in the improvement of synaptic plasticity, reduction of Aβ, and restoration of phosphatidylinositol‐3‐kinase/protein kinase B/glycogen synthase kinase‐3beta (PI3K/AKT/GSK3β) cell signaling in the hippocampus, and hence, it may be used for the treatment of AD (Wang, Han, et al., 2020).

### 6.4 Other interventions

Bmal1 gene expression in skeletal muscle was found to elevate after nearly three months of exercise intervention, along with enhanced peripheral insulin sensitivity and reduced BMI and body weight, demonstrating that metabolic benefits from long‐term exercise are partly mediated by the clock molecule Bmal1 (Erickson et al., 2020). A normal chow‐fed, ad libitum, circadian mutant mice (including mice with liver‐specific KO of *Bmal1*) developed some aspects of metabolic dysfunction that were exacerbated with age, while the time‐restricted feeding (TRF) prevented the whole‐body fat accumulation and serum hyperlipidemia, suggesting that TRF contributes to sustaining metabolic health in the condition of circadian rhythm/molecular clock defects such as *Bmal1* deletion (Chaix et al., 2019). Another study found that TRF was related to increased Bmal1 mRNA levels in the liver and improved metabolic health (Regmi et al., 2021). Adiponectin, an adipocyte‐derived hormone, ameliorates the aberrant expression of the Bmal1 mRNA/protein induced by Aβ31‐35 via inhibition of GSK3β activity, and exogenous administration of adiponectin may be a promising treatment for AD (Yuan et al., 2021). NAD+ deficiency in diverse tissues with age contributes to the progress of multiple age‐related diseases such as T2DM, AD, vascular dysfunction (de Picciotto et al., 2016), and cerebral ischemia. Accumulating evidence suggests that supplementation with nicotinamide mononucleotide (NMN) or nicotinamide riboside (NR) to increase NAD+ levels is a promising therapy for diverse aging‐related diseases (Hong et al., 2020). Recently, Zhu et al. found that increased cytosolic NAD+ could restore hypoxic cell proliferation and myofiber formation in Bmal1‐deficient myoblasts (Zhu et al., 2022). Hence, exercise, TRF, adiponectin, and supplementation with NMN and NR might also be promising treatment strategies for aging‐related diseases that are dependent on the modulation of Bmal1.

## 7 CONCLUSIONS AND FUTURE DIRECTIONS

Mounting evidence supports the existence of a significant interaction between Bmal1 deficiency/abnormality and aging‐related diseases. Some mechanisms related to Bmal1 deficiency in AD and associated aging‐related diseases (Figures 2 and 3) and interventions have been reviewed in the present study. Future studies are required to further understand the physiological intricacies of Bmal1 in the context of aging, with the following main to‐be‐explored aspects: (1) the signaling pathway network of Bmal1 mechanisms in aging‐related diseases needs to be established, (2) several studies have shown that Bmal1 deficiency is involved in the occurrence and development of many other diseases not discussed in this review such as dilated cardiomyopathy (Lefta et al., 2012; Li, Li, et al., 2020) and cancer (Stokes et al., 2021; Wang et al., 2019). Therefore, the relationship of Bmal1 with other key diseases remains to be explored, (3) third, the effects of different Bmal1‐modulating interventions on aging‐related diseases have been analyzed in animal and cell culture model studies. Patients‐oriented studies are lacking, and hence, interventions with sufficient evidence of efficacy should be selected to confirm their therapeutic potential using randomized controlled trials.

## ACKNOWLEDGMENTS

Special thanks to Hao Duan for his assistance with some image drawings.

## AUTHOR CONTRIBUTIONS

RF and YY drafted the manuscript. XP, LX, KD, DM, WX, XS, SZ, JC, XY, and YY revised the manuscript. All authors have read and agreed to the published version of the manuscript.

## FUNDING INFORMATION

This work was funded by grants from the National Nature Science Foundation of China (Grant number: 81974114) and the Jie Chu Jing Ying foundation (grant number 2018076).

## CONFLICT OF INTEREST

The authors have no conflicts of interest to disclose.

## Data Availability

No data were created or analyzed in this study. Data sharing is not applicable to this review article.
